# Correction: Molybdenum chloride double perovskites: dimensionality control of optical and magnetic properties

**DOI:** 10.1039/d3sc90092d

**Published:** 2023-05-23

**Authors:** Devesh Chandra Binwal, Prashurya Pritam Mudoi, Debendra Prasad Panda, Pratap Vishnoi

**Affiliations:** a New Chemistry Unit, International Centre for Materials Science, School of Advanced Materials, Jawaharlal Nehru Centre for Advanced Scientific Research Jakkur P.O. Bangalore-560064 India pvishnoi@jncasr.ac.in; b School of Advanced Materials, Chemistry and Physics of Materials Unit, Jawaharlal Nehru Centre for Advanced Scientific Research (JNCASR) Jakkur P. O. Bangalore 560064 India

## Abstract

Correction for ‘Molybdenum chloride double perovskites: dimensionality control of optical and magnetic properties’ by Devesh Chandra Binwal *et al.*, *Chem. Sci.*, 2023, **14**, 3982–3989, https://doi.org/10.1039/D3SC00132F.

The authors regret that Fig. 3d in the original article requires correction. On page 3985 of the original article, the heat flow unit on the *y*-axis of Fig. 3d is incorrect. The unit should be mW instead of W/g. The amended version of Fig. 3 is shown below.

**Fig. 1:**
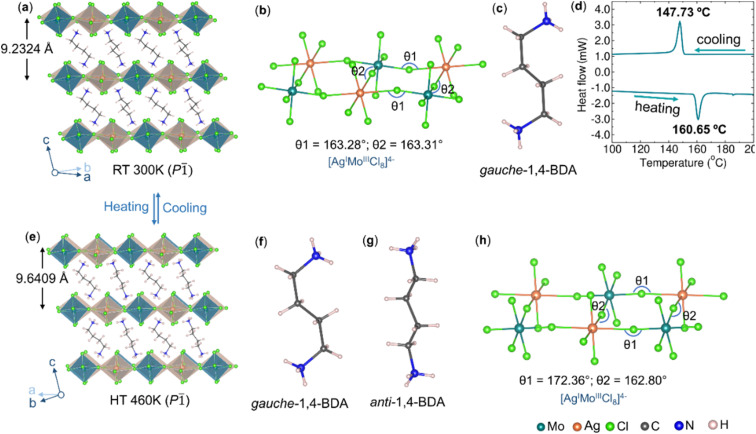
(a) Room temperature SCXRD structure of (1,4-BDA)_2_AgMoCl_8_. (b) Corresponding ball and stick models for the perovskite layer showing octahedral connectivity between [AgCl_6_]^5−^ and [MoCl_6_]^3−^ units. (c) 1,4-BDA spacer in a *gauche* conformation. (d) DSC curve of (1,4-BDA)_2_AgMoCl_8_. (e) SCXRD structure of (1,4-BDA)_2_AgMoCl_8_ at 460 K. (f and g) 1,4-BDA spacer in *gauche* and *anti* conformations. (h) Ball and stick models for the perovskite layer in the 460 K structure, showing reduced octahedral tilting than that of the room temperature structure.

The Royal Society of Chemistry apologises for these errors and any consequent inconvenience to authors and readers.

## Supplementary Material

